# Senolytic and senomorphic secondary metabolites as therapeutic agents in *Drosophila melanogaster* models of Parkinson’s disease

**DOI:** 10.3389/fneur.2023.1271941

**Published:** 2023-09-28

**Authors:** Sean J. Miller, Rayyan Y. Darji, Sami Walaieh, Jhemerial A. Lewis, Robert Logan

**Affiliations:** ^1^Department of Ophthalmology and Visual Science, Yale University School of Medicine, New Haven, CT, United States; ^2^Department of Biology, Eastern Nazarene College, Quincy, MA, United States

**Keywords:** senescence, Parkinson’s disease, *Drosophila*, secondary metabolites, neuroprotection

## Abstract

*Drosophila melanogaster* is a valuable model organism for a wide range of biological exploration. The well-known advantages of *D. melanogaster* include its relatively simple biology, the ease with which it is genetically modified, the relatively low financial and time costs associated with their short gestation and life cycles, and the large number of offspring they produce per generation. *D. melanogaster* has facilitated the discovery of many significant insights into the pathology of Parkinson’s disease (PD) and has served as an excellent preclinical model of PD-related therapeutic discovery. In this review, we provide an overview of the major *D. melanogaster* models of PD, each of which provide unique insights into PD-relevant pathology and therapeutic targets. These models are discussed in the context of their past, current, and future potential use for studying the utility of secondary metabolites as therapeutic agents in PD. Over the last decade, senolytics have garnered an exponential interest in their ability to mitigate a broad spectrum of diseases, including PD. Therefore, an emphasis is placed on the senolytic and senomorphic properties of secondary metabolites. It is expected that *D. melanogaster* will continue to be critical in the effort to understand and improve treatment of PD, including their involvement in translational studies focused on secondary metabolites.

## 1. Introduction

Parkinson’s disease (PD) is a neurodegenerative disorder that affects dopaminergic neurons in the substantia nigra pars compacta (SNpc) of the central nervous system (CNS). A key component in the development of PD is the formation of Lewy bodies (1). These are insoluble aggregates of misfolded alpha-synuclein (α-Syn) protein fibrils, among other components. Lewy bodies accumulate within neurons and contribute to their death. The degeneration of SNpc dopaminergic neurons leads to a pathologic reduction of dopamine at the striatum. As a result, individuals with PD experience motor symptoms like resting tremors, slowness of movement (bradykinesia), erratic and writhing movements of the face, arms, legs, or trunk (dyskinesia), rigidity, stiffness, postural instability, and compromised balance (2). Additionally, people with PD have non-motor symptoms, including cognitive changes, sleep disorders, autonomic dysfunction, gastrointestinal issues, and mood disorders (3).

PD is thought to be caused by a combination of genetic and environmental factors, with only around 10% of people with PD having one of the identified genetic mutations, such as *SNCA* (Synuclein Alpha), *PINK1* (PTEN Induced Kinase 1), and *PRKN* (Parkin RBR E3 ubiquitin protein ligase) (4). A study by Goldman et al. investigating the genetic contribution to PD risk using concordance rates in monozygotic and dizygotic twins found the hereditability of PD to be around 27%, further suggesting that nongenetic factors may be the more predominant sources of PD risk (5).

In addition to genetic contributions, environmental toxins have been associated with PD, including pesticides such as rotenone and paraquat, can enter the human body through various routes, including inhalation, ingestion of residues in food and water, and dermal exposure. Epidemiologic studies have commonly found that PD is correlated with farming and rural environments due to exposure to pesticides and herbicides (6, 7).

Medical management of PD is a significant challenge, as existing therapies have limited effectiveness and undesirable side effects. For example, dyskinesia is a common side effect of long-term use of levodopa, the most common medication used to manage PD (8). This form of dyskinesia, known as levodopa-induced dyskinesia (LID), can make it difficult for an individual to perform routine tasks of daily living. Therefore, alternative therapeutic agents that can effectively target the underlying mechanisms of PD without causing significant adverse effects are greatly needed and highly sought after. One approach to addressing this need is exploring the therapeutic potential of secondary metabolites in pre-clinical *Drosophila melanogaster* models of PD.

*D. melanogaster*, colloquially referred to as the fruit fly, is a valued and versatile model organism. For example, it exhibits a relatively simple biology with a well-characterized genome, anatomy, and molecular pathways that are also well-conversed within higher organisms, including humans (9). Their biological simplicity facilitates relatively easy and precise genetic manipulation. Therefore, they have been indispensable in the study of genetics, disease mechanisms, and the identification of novel therapeutic targets. Finally, *D. melanogaster* have short gestation periods and a high reproductive capacity, enabling rapid experimental turnover, large-scale genetic screens, and developmental research (10). This feature is particularly advantageous for studying the effects of genetic alterations or environmental factors over successive generations. Practically, it also offers cost-effectiveness, with relatively low financial and time costs associated with maintenance and experimentation (11, 12).

Secondary metabolites, sometimes referred to as phytochemicals or bioactive compounds, are natural products extracted from plants. These metabolites are not essential for the plant’s growth, development, or reproduction. Instead, they are produced by plants as a defense mechanism against external threats such as UV radiation, predators, and pathogenic microorganisms (13). Secondary metabolites have been compiled into four groups based on their chemical structure: terpenoids, phenylpropanoids, polyketides, and alkaloids (14). Examples of terpenoids include limonene from the rind of lemons and oranges, menthol from peppermint, and taxol from the Pacific yew tree, which is used in chemotherapy. Phenylpropanoids (phenolics) are compounds such as curcumin from turmeric, resveratrol from grapes, and capsaicin from chili peppers (15). Polyketides are complex and usually have antibiotic or anticancer properties. Examples include the antibiotic erythromycin and the chemotherapeutic agent doxorubicin. Lovastatin is a polyketide used for lowering cholesterol (16). Examples of the fourth classification, alkaloids, include morphine from opioid poppies, quinine from Cinchona trees, and caffeine from coffee beans (17).

## 2. Overview of relevant *Drosophila melanogaster* models of PD

There are two broad categories of *D. melanogaster* models of PD – those made through genetic manipulation and those that are toxin-induced. In genetic models, PD-associated genes can be knocked down or overexpressed to highlight their effects on phenotype and risk. Toxin models involve either an acute dose or chronic dosing. Sublethal, chronic dosing closer reflects the natural progressive course of neurodegeneration and is not as likely to prematurely kill the flies when compared to the more convenient acute dose method. Toxin models are better suited for studying the potential environmental impacts on PD risk and development, whereas genetic models provide valuable insights into familial cases. In both genetic and toxin models, the role of oxidative stress and mitochondrial dysfunction in dopaminergic neuronal degeneration is notable.

### 2.1. SNCA

*D. melanogaster* models of PD have been able to faithfully recreate some of the key features of the disease, including the formation of α-Syn aggregates into Lewy bodies and the loss of dopaminergic neurons. Among the pathological conditions that can act as an impetus for α-synucleinopathy is the overexpression of α-Syn itself. However, *D. melanogaster* do not have a homolog for the human gene that encodes for α-Syn, namely, *SNCA* (18). However, induced transgenic overexpression of human α-Syn in *D. melanogaster* has been achieved using various approaches, including the Gal4-upstream activating system, which allows for tissue-specific and inducible expression of transgenes (18).

Transgenic models that overexpress human α-Syn provide opportunities to study Lewy body formation and potential therapeutic interventions. The *D. melanogaster* transgenic α-Syn model has demonstrated that α-Syn overexpression leads to progressive locomotor impairments, including reduced climbing ability and impaired flight, reminiscent of motor deficits observed in PD patients (19, 20). Furthermore, α-Syn overexpression in *D. melanogaster* leads to the formation of intracellular aggregates and the loss of dopaminergic neurons, mimicking the neurodegenerative process observed in PD (20). At the molecular level, these models have shown that α-Syn-induced neurodegeneration involves oxidative stress, mitochondrial dysfunction, impaired protein degradation pathways, and synaptic dysfunction (21). Furthermore, genetic modifiers that enhance or suppress α-Syn-induced toxicity have been identified in *D. melanogaster*, which underscores the intricate genetic interactions of PD pathology and highlights potential therapeutic targets (22).

### 2.2. *PINK1* and *PRKN*

Genetic mutations in *PINK1* and *PRKN* are the most commonly known causes of early-onset familial PD, and these mutations are also linked to sporadic forms of PD (23, 24). Both genes have been identified as critical regulators of mitochondrial quality and function within the same biological pathways (25). When a mutation in either of these genes stops them from functioning normally, mitochondria become dysfunctional and fail to be cleared. Significantly, manipulating these genes in *D. melanogaster* mimics the cellular pathophysiology of PD.

For example, loss-of-function mutations in *PINK1*, by either disrupting gene expression or protein function, have been extensively studied using *D. melanogaster* as a model organism. Indeed, the first *in vivo* report of *PINK1* manipulation was conducted in *D. melanogaster* (26). This seminal study and other early work revealed that a loss of the *D. melanogaster PINK1* homolog (CG4523) results in fragmented mitochondria, reduced ATP levels, muscle degeneration, locomotor deficits, dopaminergic neuronal loss, male sterility, and increased sensitivity to oxidative stress (27, 28). These effects are reversible by the expression of human *PINK1* or with overexpression of *PRKN* (26). Furthermore, *D. melanogaster* that overexpress *PINK1* demonstrate improved mitochondrial function, enhanced resistance to oxidative stress, preserved dopaminergic neuronal survival, and a longer lifespan compared to wild type controls (29–31). The use of *PINK1* and *PRKN* genetic models in *D. melanogaster* has yielded several key findings regarding the molecular mechanisms of PD pathogenesis, such as the importance of maintaining mitochondrial homeostasis and protecting against cellular stress.

Loss-of-function mutations in *PRKN* (a.k.a. *PARK2*) in *D. melanogaster*, either by knockout or RNAi knockdown, leads to the disruption of mitochondrial function, increased cellular sensitivity to oxidative stress, and phenotypical locomotor defects, similar to the clinical features of PD (32, 33). Furthermore, studies using *PRKN* overexpression in *D. melanogaster* have elucidated the protective effects of Parkin (the protein product of *PRKN*) on mitochondrial function and cell viability. For example, *PRKN* overexpression enhances mitochondrial quality control mechanisms, such as mitophagy, promotes the elimination of damaged mitochondria, delays aging, extends the lifespan, and protects against senescence in *D. melanogaster* (34). Moreover, *PRKN* overexpression in *D. melanogaster* can rescue mitochondrial defects caused by other genetic mutations or environmental stressors (35). These findings suggest that Parkin plays a crucial role in maintaining mitochondrial homeostasis and protecting against the neurodegenerative processes in PD.

The aforementioned genetic models have been chosen for discussion because they have been used to study the effects of senolytic secondary messengers. However, there are other important *D. melanogaster* genetic models of PD. For example, *D. melanogaster* that harbor mutations in either the dominant genes for glucocerebrosidase (GBA) or vacuolar protein sorting 35 (VPS35) or in the recessive gene DJ-1 produce phenotypes similar to those observed in idiopathic PD (36). It is important to note some genes implicated in PD pathology can play different roles in dopaminergic neuron degeneration depending on the specifics of the model used (37). Therefore, it is critical to be mindful that while neurodegeneration might be morphologically and behaviorally identical in many distinct experimental contexts, the underlying genetic pathways and cellular programs might differ.

### 2.3. Rotenone

A commonly used toxin for *D. melanogaster* models of PD is rotenone, a naturally occurring pesticide implicated in the development of sporadic PD (38). Rotenone has lipophilic properties that allow it to readily cross the blood–brain barrier (BBB) (39). The mechanism of action of rotenone involves inhibiting the function of complex I in the mitochondrial electron transport chain, leading to pathologically high oxidative stress, neuronal dysfunction, and, eventually, cell death (40, 41). A study by Sherer et al. demonstrated that chronic rotenone exposure to human neuroblastoma cells causes complex I inhibition and may lead to the accumulation and aggregation of α-synuclein (42). Exposure to rotenone has also been shown *in vitro* to lead to endoplasmic reticulum stress and to be associated with the unfolded protein response (43). The chronic administration of rotenone to *D. melanogaster* results in the selective loss of dopaminergic neurons, the formation of protein aggregates similar to Lewy bodies, and locomotor deficits (44, 45). Therefore, this model exhibits all the classic pathological features of PD.

### 2.4. Paraquat

Paraquat (1,1′-dimethyl-4,4′-bipyridinium dichloride) is an herbicide with sweeping, nonspecific weedkilling properties. It has been used in more than 100 countries to protect crops such as cotton, cocoa, tobacco, soybean, rice, and others (46). In the context of its intended agricultural use, paraquat is generally considered safe when applied following recommended guidelines and safety precautions (47). However, it can easily seep into groundwater and has been detected in harvested fruits and vegetables, which raises environmental and food safety concerns (47). Paraquat has also been shown to be highly toxic for fish, algae, and mammals, even at low levels (46).

The toxicity of paraquat is attributed primarily to its role in producing noxiously high levels of reactive oxygen species (ROS). Through redox-cycling reactions, paraquat is taken up by mitochondria and reduced by nicotinamide adenine dinucleotide phosphate (NADPH) to produce the highly reactive superoxide (O_2_^−^) anion (48–50). In addition, O_2_^−^ can subsequently lead to a rise in toxic ROS, such as hydrogen peroxide (H_2_O_2_) and hydroxyl radicals (HO•), which cause extensive oxidative damage to cellular components such as DNA, lipids, and proteins. Paraquat exposure has also been associated with damage to complex I of the electron transport chain, which also increases ROS burden (51). Furthermore, studies have shown that paraquat increases the levels of p53 protein and its downstream target genes, such as the pro-apoptotic protein, Bax (51). Finally, paraquat induces cellular senescence, a critical component of PD pathology (52, 53). Oxidative stress, its associated cellular dysfunction, senescence, and apoptosis are key pathological hallmarks of PD. Although environmental exposure to paraquat has been widely linked to an increased risk of PD, some epidemiological studies do not show a correlative relationship (54, 55).

*D. melanogaster* treated with paraquat experience elevated oxidative stress-induced lipid peroxidation in the brain, evidenced by increased quantities of the end product of lipid peroxidation, namely malondialdehyde (56). In addition, the quantity of O_2_^−^ and H_2_O_2_ in the brains of *D. melanogaster* dramatically increases between 2 and 4-fold in response to paraquat-induced oxidative stress (56, 57). Concurrently, the activity of antioxidant enzymes, such as superoxide dismutase (SOD), is significantly reduced in paraquat-treated *D. melanogaster* models of PD, which exacerbates the rate and extent of cellular damage caused by paraquat-induced oxidative stress (56). However, some studies show the opposite trend of SOD activity increasing in paraquat-treated *D. melanogaster*, presumably as a defense mechanism (57). Indeed, SOD activity was shown to be elevated in the substantia nigra and basal ganglia of postmortem brains of PD patients (58). Similarly, the transcription factor nuclear factor erythroid 2-related factor 2 (Nrf2) is a critical regulator of the cellular response to oxidative stress (59). Nrf2 mRNA expression increases in paraquat-treated *D. melanogaster* just as it does in leukocytes and dopaminergic neurons of PD patients (60, 61). These results suggest that despite an attempt by cells to protect themselves from oxidative stress, disease ensues when their efforts are insufficient. Beyond oxidative stress, apoptosis, and mitochondrial dysfunction, *D. melanogaster* paraquat models replicate other PD-relevant pathology, such as elevated nitrosative stress, impaired dopamine metabolism, reduced brain-derived neurotrophic factor, and heightened endoplasmic reticulum stress (62).

### 2.5. MPTP and 6-OHDA

1-methyl-4-phenyl-1,2,3,6-tetrahydropyridine (MPTP) is another commonly used toxin for mimicking PD pathology and behavior, as it specifically targets dopaminergic neurons in the substantia nigra. MPTP is converted *in vivo* to 1-methyl-4-phenylpyridinium (MPP+) by monoamine oxidase-B. MPP+ is then taken up by dopaminergic neurons via the dopamine transporter and interferes with mitochondrial function, leading to cell death. Although not as commonly used as paraquat or rotenone, MPTP has successfully been used in *D. melanogaster* models of PD to study the dopaminergic neuroprotective properties of resveratrol and trans-astaxanthin (63, 64). 6-Hydroxydopamine (6-OHDA) is another commonly used toxin to induce PD-like pathology. Although widely used in rodent models, it has rarely been used in *D. melanogaster* models. 6-OHDA cannot pass the BBB, therefore necessitating an injection to the brain. Due to *D. melanogaster* anatomy, this is not a favorable model (65). The mechanism of action of 6-OHDA involves entering catecholaminergic neurons through dopamine membrane transporters (DAT) or noradrenaline membrane transporters (NAT) and accumulating intracellularly (66). The metabolism of 6-OHDA by monoamine oxidase-A yields H_2_O_2_, triggers the generation of ROS, and inhibits complex I of the electron transport chain (67).

There are other toxins used to produce PD-related phenotypes. For example, *D. melanogaster* exposure to iron (Fe) induced PD-like motor and non-motor symptoms, which were attenuated by the co-administration of hesperidin, a citrus flavonoid (68). Hesperidin was more effective than L-DOPA at protecting against motor coordination, memory, and anxiety deficits, in addition to reducing the concentration of caudal Fe (68).

## 3. Senolytic and senomorphic secondary metabolites in *Drosophila melanogaster* models of PD

Senescence is a complex phenomenon of irreversible growth arrest in cells. It plays a crucial role in various stress responses, aging processes, and chronic disease. Multiple factors contribute to the development of senescence, such as DNA damage, telomere shortening, oncogenesis, oxidative stress, and inflammation (69, 70). The accumulation of senescent cells in tissues is both a consequence and a contributing factor of age-related pathologies, therefore feeding into a positive feedback loop of increasing pathology (53). For instance, senescent cells secrete a molecular cocktail of proinflammatory cytokines, chemokines, and tissue-damaging proteases known collectively as the senescence-associated secretory phenotype (SASP). Although SASP is an initial protective mechanism against foreign entities, it can also exacerbate chronic inflammation and contribute to tissue dysfunction (53, 71).

Secondary metabolites are organic compounds that are not directly involved in the growth, development, and reproduction of an organism. Instead, they play important roles in various physiological processes such as defense against predators, competition for resources, and communication among organisms (72). Secondary metabolic pathways are also diverse within and across organisms. Often, they are enzymatically derived from primary metabolites, intermediates in primary metabolism, or are unique metabolic precursors. Plants are a common source of secondary metabolites used for health and wellness. The biosynthesis and storage of plant secondary metabolites occur in structures such as vacuoles, glandular trichomes, cuticles, and oil cells (73).

There are several secondary metabolites that have demonstrated potential in treating PD. Capsaicin, an active compound from chili peppers, was seen to protect dopaminergic neurons in PD mouse models by supporting mitochondrial function and inhibiting neuroinflammation (74). Berberine, an isoquinoline alkaloid from Chinese herbs, has shown potential in PD treatment through its antioxidant, anti-inflammatory, and anti-apoptotic effects (75). Caffeine, commonly contained in coffee and tea, has been studied for its neuroprotective effects in modulating adenosine receptors and reducing neuroinflammation, potentially providing protection against PD progression (76). The naturally produced alkaloid nicotine, found in small quantities in certain foods, has exhibited neuroprotective effects on dopaminergic neurons to modulate neurotransmitter systems affected in PD (77). Each of these compounds has shown promise in preclinical studies using *D. melanogaster* models, and some have moved to clinical trials (78–80).

Senolytic secondary metabolites selectively target senescent cells for induced apoptosis or programmed death, ultimately leading to their clearance from tissues and possible replacement with healthy cells (81). Senolytics have shown exciting promise in pre-clinical and clinical studies for their ability to enhance tissue regenerative capacity and alleviate age-related pathologies (82). While the relatively nascent study of senolytic secondary metabolites in the treatment of neurodegeneration is still evolving, several bioactive compounds have shown encouraging neuroprotective properties through their senolytic activity.

The mechanism of action and efficacy of senolytic secondary metabolites vary depending on the metabolite and cellular environment (81, 83, 84). For example, in response to cellular stress, some senolytic secondary metabolites will activate either the intracellular p53 or P16^INK4A^ pathways, leading to a senescent cellular state (85). Other secondary metabolites act through senescence-associated vulnerabilities, exploiting specific characteristics or dependencies of senescent cells. These vulnerabilities include altered metabolic profiles, increased reliance on anti-apoptotic proteins, or dysregulated stress response pathways (86). By targeting these vulnerabilities, senolytic secondary metabolites can induce senescent cell death while sparing healthy cells. Furthermore, secondary metabolites often possess anti-inflammatory and antioxidant properties, which can contribute to their overall therapeutic effects. By reducing the inflammation and oxidative stress associated with SASP, these compounds can potentially attenuate the damaging effects of senescent cells on surrounding tissues without being true senolytics and are, instead, senomorphics (87–90).

Senomorphic secondary metabolites mitigate the detrimental effects associated with senescent cells rather than selectively eradicating them. Compared to senolytics, they are a recently recognized behavioral class of cells that has not received as much attention. For example, a default PubMed search for “senolytic” returns about 1,165 items – beginning in 2014. In contrast, a default PubMed search for “senomorphic” returns about 75 items – beginning in 2019. However, there is a growing body of evidence that is illuminating their mechanisms of curtailing the pro-inflammatory SASP. Therefore, they have emerged as promising candidates for novel therapeutic interventions in age-related diseases, including cancer, neurodegenerative disorders, and cardiovascular diseases. By harnessing the power of senescence modulation, senomorphic secondary metabolites offer a nuanced approach to counteract the detrimental effects of cellular senescence, promoting tissue rejuvenation and potentially extending healthspan. Further research into the mechanisms underlying the senomorphic effects of these compounds is warranted.

There are many classifications and subclasses of secondary metabolites based on their chemical architecture and biosynthetic pathways. The currently known senolytic secondary metabolites are broadly categorized as either phenolic compounds or alkaloid compounds (91).

Phenolics have one or more aromatic rings with one or more hydroxyl groups and are products of phenylpropanoid metabolism, downstream of the shikimic acid and malonic acid pathways (92). The flavonoid subclass of phenolic compounds is the most abundant type found in fruits and vegetables and contains six subfamilies: flavones, isoflavones, flavonols, flavanones, flavan-3-ols, and anthocyanidins (92).

Alkaloids are characterized by a nitrogen-containing heterocyclic ring and are derived from several metabolic pathways (93). For example, the shikimate pathway produces aromatic amino acids that serve as metabolic precursors to many alkaloids. The shikimate pathway also directly produces some alkaloids in addition to the terpenoid pathway. The polyketide pathway, like the shikimate pathway, provides many alkaloid precursors (94).

### 3.1. Quercetin

Quercetin is a senolytic phenolic compound of the flavonoid class, flavonol subfamily. Flavonols are the most ubiquitous flavonoid and have high concentrations in onions, grapes, cherries, apples, citrus fruits, and tomatoes (95). Since quercetin is the main flavonol representative, it has been extensively studied for its health-related benefits, including its antioxidant, anti-inflammatory, anti-microbial, and senolytic properties (53, 96–98). Quercetin’s senolytic activity includes inducing apoptosis of senescent cells, mitigating the effects of SASP, providing a robust antioxidant capacity either directly or by increasing glutathione levels, and activating autography to clear senescent cells (95, 99, 100). Therefore, it demonstrates both pure senolytic and senomorphic properties.

Silkworms have been used for medicinal purposes for more than 5,000 years. Silkworm extract has been used in treating diabetes, liver disease, and hypertension, even demonstrating the ability to regenerate axons and promote Schwann cell proliferation (101). Mature silkworms can be steamed, freeze-dried, and then processed into the ingestible Hongjam (102). Hongjam contains high levels of flavonoids, the most abundant of which is quercetin (103). In a recent study, normal *D. melanogaster* feed was supplemented with golden-silkworm Hongjam (GSHJ), which extended their lifespan and the length of time they voluntarily engaged in locomotion (102). Furthermore, GSHJ was protective against the PD phenotype of rotenone-treated *D. melanogaster*. For example, it prevents the onset and progression of rotenone-induced motor control deficit (102). The neuroprotective mechanisms of Hongjam are attributed in part to its enhancement of the electron transport chain activity (mitochondria complexes I through IV). Additionally, consumption of the GSHJ supplemented diet suppressed autophagy signaling and UPR signaling, both of which are necessary to establish senescence (104–107). Hongjam also has anti-inflammatory properties, reducing cytokine levels (108).

Blue light irradiation is toxic to *D. melanogaster* by increasing oxidative stress and accelerating their aging (109). In a recent study, *D. melanogaster* exposed to blue light irradiation and quercetin treatment experienced a quercetin-mediated reduction of senescent cells, decreased protein content in males, and decreased lipid content in females (110). Behaviorally, quercetin improved lifespan and extended the time the flies voluntarily engaged in motor activity, which the authors interpreted as the health span. Quercetin also improved their heat stress survival, increased male activity levels, and boosted female egg production (110). Although blue light caused oxidative stress, senescence, and pre-mature aging in *D. melanogaster*, it did not specifically lead to a PD model. However, neurodegeneration, at least in the early stages of the disease, shares many of the physiological changes associated with increased oxidative stress, inflammation, and senescence.

Similarly, *D. melanogaster* that have been treated with H_2_O_2_ also experience pathological levels of oxidative stress, disrupted redox homeostasis, and exhibit signs of general stress such as significantly elevated levels of Upd1, a homolog of IL-6 (111). Additionally, H_2_O_2_ treatment of *D. melanogaster* increases the expression of stress-related genes, the synthesis of heat shock protein-70 (Hsp70), and neuronal apoptosis caused by nitric oxide (NO) (111, 112). Quercetin treatment of *D. melanogaster* exposed to H_2_O_2_ nearly completely neutralizes the increase in Hsp70 and Upd1, in addition to preserving performance on the negative geotaxis assay (111). Furthermore, quercetin mitigates H_2_O_2_-driven increased levels of protein carbonyls and thiobarbituric reactive substances, which serve as general indicators of oxidative stress. Accordingly, quercetin rescues the reduced level of antioxidants superoxide dismutase, glutathione, and catalase generated by H_2_O_2_ exposure (111). As with blue light, H_2_O_2_ does not specifically lead to a PD model of *D. melanogaster*. However, these results have translational value with neurodegeneration.

*D. melanogaster* exposed to acute and sub-acute doses of paraquat have also been used to investigate the PD-specific neuroprotective properties of quercetin. Either H_2_O_2_ or paraquat exposure can be sufficient to induce cellular senescence related to PD pathology. The mechanism of action for these toxins includes the initiation of DNA damage, compromised proteostasis, neuroinflammation, and oxidative stress (52, 113–115). The neuroprotective capabilities of the secondary metabolites 4-hydroxyisophthalic acid, ellagic acid, and quercetin, in addition to the primary metabolite nicotinamide – a precursor to the coenzyme nicotinamide adenine dinucleotide – have been compared (116, 117). All four of these compounds improved the survival rates of *D. melanogaster* in a dose-dependent manner after the flies had been exposed to an acute, high dose of paraquat. However, males had a better overall survival rate per antioxidant. Furthermore, the ability of quercetin to preserve mortality was greater in males (116). Among the metabolites, nicotinamide had the greatest effect on females (116). In the sub-acute paraquat exposure model, all bioactive compounds improved performance on the negative geotaxis assay, reduced ROS levels, reduced lipid peroxidization levels, protected against glutathione depletion, and the activity of superoxide dismutase and catalase. There was no appreciable difference in the benefits of 4-hydroxyisophthalic acid, quercetin, and nicotinamide, where ellagic acid was overall the least protective (116).

The mechanisms of quercetin’s neuroprotective properties have been further studied beyond *D. melanogaster* PD models. For example, in 6-OHDA treated rats and PC12 cell culture, quercetin is shown to be a powerful antioxidant, can upregulate PINK1 and Parkin, protects mitochondria from damage, and inhibits α -Syn accumulation (118). Furthermore, quercetin disaggregates α -Syn fibrils through covalent bonding with α -Syn fibrils rather than through antioxidant activity (119). Quercetin covalently bonds to α -Syn fibrils, oligomers, or monomers, which increases their hydrophilicity. Therefore, the fibrillation of α -Syn and subsequent aggregation is inhibited (119). Excitatory cells of the cerebellum that have been treated with H_2_O_2_ experience nuclear translation of Nrf2 and increased expression of glutathione when exposed to quercetin (120). Finally, *in silico* molecular docking experiments have revealed that the active sites of monoamine oxidase-B stably interact with quercetin, suggesting that quercetin could prevent increased ROS production and oxidative stress associated with the upregulation of monoamine oxidase-B in PD (121).

### 3.2. Fisetin

Fisetin is a senolytic phenolic compound of the flavonoid class, flavonol subfamily. It is one of the most abundantly available and studied flavonols (122). Fisetin is found in various fruits and vegetables and is predominant in strawberries, apples, and onions. Like all flavonoids, its biosynthesis begins with the shikimate pathway, which feeds into the phenylpropanoid pathway to produce phenylalanine, and then begins flavonoid biosynthesis after converting to 4-coumaroyl-CoA (123). Fisetin has been extensively studied for its wide-ranging medicinal applications, including its senolytic effects (124).

In a study by Jhonsa et al., a paraquat-treated *D. melanogaster* model of PD experienced a diminished lifespan and negative geotaxis assay performance in a dose-dependent manner, spanning from 5 mM to 15 mM (125). At the 15 mM dose of paraquat, mortality was 100% within 24 h. L-DOPA treatment at 2 mg/mL improved survival among the 15 mM paraquat-treated *D. melanogaster* to more than 35% at the 24-h mark. However, treatment of 2.8 mg/mL fisetin boosted the survival rate to more than 45%. Fisetin also rescued locomotor deficits on the negative geotaxis assay to about 90% of the control, whereas L-DOPA improved performance to only about 75% of the control. Fisetin treatment also rescued glutathione, catalase, and superoxide dismutase quantity and the enzymatic activity of acetylcholine esterase (AChE). Consequently, fisetin facilitated reduced ROS accumulation compared to L-DOPA treatment and control. L-DOPA was not as effective as fisetin in all parameters assayed, except for rescuing AChE activity, where it dramatically outperformed fisetin (125).

In a related study, a cell culture model of PD (human neuroblastoma SH-SY5Y cells treated with 6-OHDA) experienced increased expression of several genes related to oxidative stress. However, their expression was suppressed by fisetin in a dose-dependent manner (126). Furthermore, fisetin decreased the 6-OHDA mediated increase in ROS production, cell death driven by apoptosis, and cell death driven by P13K–Akt signaling (126). Unfortunately, outside of the therapeutic range, fisten is cytotoxic at concentrations of 50 μ M and 100 μ M, whereas 6-OHDA was severely cytotoxic at only 10 μ M. At 50 μ M of either 6-OHDA or fisetin, lactate dehydrogenase levels rose significantly, indicating cell damage (126).

Fisetin’s antioxidant, neuroprotective, and senolytic effects have been demonstrated in other aged and neurodegenerative models. For example, *D. melanogaster* ALS models that express hSOD1^G85R^ (the mutated version of human SOD1), develop rapid and late-onset ALS symptoms (127). Compared to the control, *D. melanogaster* hSOD1^G85R^ that received fisetin treatment experienced longer lifespan, longer health span, curbed ROS production, stabilized redox homeostasis, reduced the levels of wild type and mutated hSOD1 protein, and improved motor skills (127). A part of fisetin’s mechanism of action is to increase the expression of the extracellular signal-regulated kinase (ERK) pathway, which upregulates a variety of genes in antioxidant systems. This action of fisetin has also been shown in AD mouse models, *D. melanogaster* and mouse models of Huntington’s disease, and other ALS models (128–130). Finally, in naturally aged mice and in mouse models of progeroid syndrome, which exhibit tumor suppressant and senescent characteristics due to the addition of a p16^INK4a^-luciferase reporter, fisetin was the most potent senolytic out of nine other tested flavonoids (124).

### 3.3. Curcumin

Curcumin is a senolytic phenolic compound of the polyphenol class, curcuminoid subfamily. Curcumin is responsible for the distinctive yellow color of the spice turmeric, which is derived from the rhizomes of the *Curcuma longa* plant. The biosynthesis of curcumin involves the conversion of feruloyl-CoA and malonyl-CoA to feruloyldiketide-CoA via diketide-CoA synthase (131). Curcumin is then synthesized via curcumin synthase combining feruloyldiketide-CoA and feruloyl-CoA (131).

The health benefits of curcumin include potent anti-inflammatory, antioxidant, anti-apoptotic, and immunomodulatory properties, which have been observed in both animal and clinical studies (132, 133). Considerable attention has been shown to curcumin due to its anticarcinogenic, antidepressant, cardioprotective, and neuroprotective applications, among other clinical benefits (133–135). There is robust evidence that curcumin suppresses the pro-inflammatory cytokines IL-1, IL-1β, IL-4, IL-5 IL-6, IL-8, IL-10, IL-12p70, IFNγ, MCP-1, and TNF-α (133, 136, 137). It also reduces the total and differential white blood cell count, reduces iNOS mRNA levels, inhibits the COX-2 enzyme, and increases BDNF production. Furthermore, curcumin inhibits NF-κB signaling, suppresses the MAPK pathway, stimulates the BDNF/tyrosine kinase receptor B/PI3k/Akt pathway, as well as the PI3k/Akt/GSK3 pathway (133, 136–138).

Curcumin targets senescent cells for clearing. The senolytic capacity of curcumin has been demonstrated in a variety of contexts and cell types (136, 139, 140). Additionally, the curcumin analog, EF24, is a strong senolytic that induces apoptosis of senescent cells through Bal-2 proteasome degradation, independent of producing ROS (141). Other curcumin analogs have been identified, such demethoxycurcumin, bisdemethoxycurcumin, calebin A, C1, and A13 (142). Intriguingly, it has been suggested that the senolytic-related benefits of curcumin are actually senomorphic in nature, due to its activation of sirtuins and AMPK rather than directly inhibiting and removing senescent cells (143).

Sulforaphane (from broccoli seed extract) and curcumin extend the lifespan of male and female *D. melanogaster* individually. Synergistically, they extend the lifespan even greater than either alone (144). A concoction of two parts curcumin and five parts broccoli seed extract at a concentration of 0.8 g/L yielded the greatest improvement in lifespan extension of about 20 days. RNA-Seq analysis of brain tissue revealed that the phytochemical blend either upregulated or downregulated about 70 genes, which was confirmed with qPCR. Downregulated genes were the majority and had the greatest differential expression. KEGG and GO analysis of the top hits confirmed that the downregulated genes would confer longevity and altered metabolism. RNAi knockdown of the top deferentially expressed genes confirmed that they were neuroprotective against age-related dopaminergic degeneration. Furthermore, in the protocerebral posterior medial bundle, the number of dopaminergic neurons increased. Finally, RNAi knockdown of the top candidate genes preserved the lifespan of *D. melanogaster* that were treated with paraquat (144).

α -Syn misfolding and aggregation is a pathological hallmark of PD. The N-terminus is a PD mutation hotbed, the C-terminus is intrinsically disordered, and the central hydrophobic “non-amyloid-β-component” (NAC) is the domain that binds with other α -Syn proteins to self-aggregate (53). Small soluble oligomers are formed early in the aggregation cycle and are more toxic than the large, mature fibrils that accumulate in Lewy bodies (145). Therefore, preventing α -Syn is a critical strategy for mitigating α -Syn fibrillation. A recent molecular dynamics *in silico* study argues that curcumin has the capacity to destabilize soluble α -Syn oligomers by binding to the hydrophobic region to increase oligomer root-mean-squared deviation and radius of gyration, and to reduce β-sheet content and the number of backbone hydrogen bonds (146). Together, these results confirm that curcumin can biochemically prevent α -Syn fibrillation and aggregation into Lewy bodies. Previous *in vivo* and *in vitro* studies have suggested that curcumin can inhibit α -Syn aggregation (147, 148).

dUCH is a *D. melanogaster* homolog of the human UCH-L1 gene that is exclusively expressed in CNS neurons and is involved in ubiquitin-proteasome activity (149). In PD, mutated UCH-L1 colocalizes with α -Syn in Lewy bodies and enhances α -Syn aggregation (150–152). In normal physiological conditions, UCH-L1 prevents the fibrillation and aggregation of α -Syn, and also inhibits the synthesis of monoamine oxidase-B, leading to increased dopamine levels (142, 153). Knockdown of dUCH in *D. melanogaster* adults and larvae caused elevated ROS in the eye imaginal discs and dopaminergic neurons (154). Conversely, curcumin treatment reduced ROS levels 0.6-fold in eye imaginal discs and 0.5-fold in adult brains. The performance of the larval crawling assay and adult climbing assay was also negatively impacted by dUCH knockdown. Interestingly, dUCH knockdown larvae crawled more slowly than controls and exhibited tremors, reminiscent of the clinical presentation of PD. However, when treated with curcumin, dUCH knockdown larvae had greater crawling speeds than knockdown larvae without curcumin treatment. In dUCH knockdown adult flies, climbing ability decreased every consecutive day after the knockdown procedure. However, curcumin treatment provided climbing improvements at every time point studied. Furthermore, histological analysis confirmed that in larval and adult flies, dUCH knockdown reduced the number of dopaminergic neurons. However, curcumin treatment of dUCH knockdown flies resulted in preserved dopaminergic neuron quantity at both the larval and adult stages (154).

Adult male *D. melanogaster* experience dose-dependent mortality in response to rotenone exposure. Sub-acute doses of 500 μ M for 14 days resulted in a mortality rate of about 90%. However, when pre-treated with curcumin dosed at 500 μ M a day for 6 days, *D. melanogaster* mortality was reduced to about 50% (155). Negative geotaxis performance was reduced by 70% in the *D. melanogaster* treated with rotenone. However, pretreatment with curcumin for 5 days significantly restored the rotenone-induced crawling deficit in a dose-dependent manner. Rotenone exposure increased ROS and hydroperoxides, as well as reduced glutathione levels, all of which were restored with curcumin (155). Finally, the levels of dopamine and its metabolites diminish in a rotenone-treated fly, whereas curcumin pretreatment preserves them (155). Another study confirmed that curcumin was neuroprotective in the rotenone-induced *D. melanogaster* model of PD by mitigating ROS production and restraining caspase-3 and caspase-9 activity (156).

Dietary supplementation with 25 μ M, 50 μ M, and 100 μ M of curcumin significantly extended the lifespan and health span of transgenic α -Syn *D. melanogaster* in a dose-dependent manner (157, 158). In addition to promoting longevity, curcumin also reduces levels of oxidative stress, lipid peroxidation, protein carbonyl, and cell death in the brains of the transgenic α -Syn *D. melanogaster* PD models (157, 158). Transgenic LRRK2 *D. melanogaster* exposed to chronic 0.1% H_2_O_2_ treatment starting day 1 post-eclosion had a shortened lifespan, impaired climbing assay performance, diminished numbers of dopaminergic neurons, increased brain oxidized protein levels, and increased LRRK2 kinase activity compared to untreated transgenic LRRK2 *D. melanogaster*. 1 mM curcumin extended the lifespan, improved climbing assay performance, suppressed the loss of dopaminergic neurons, reduced brain oxidized proteins, and inhibited LRRK2 kinase activity in transgenic LRRK2 *D. melanogaster* that were treated with chronic 0.1% H_2_O_2_ compared to those that were not treated with chronic 0.1% H_2_O_2_ (159).

Despite the remarkable senolytic and neuroprotective abilities of curcumin, it is limited as a therapeutic agent due to its poor bioavailability. Several factors contribute to its low bioavailability. For example, curcumin is not very soluble in water, which encourages it to aggregate, resist gastrointestinal absorption, and be eliminated from the body (160). However, a slight amount gets absorbed through the gastrointestinal tract, which then undergoes significant hepatic metabolism, effectively inactivating the majority of available curcumin (160). Following hepatic metabolism, it is quickly expelled from the body through the gall bladder (161). Finally, curcumin does not evenly distribute across body tissues, nor does it easily cross the BBB (162, 163).

Therefore, recent attempts to exploit curcumin’s neuroprotective capacity have focused on nanodelivery strategies, including polysaccharide nanoparticles, silica nanoparticles, nanosuspensions, carbon nanotubes, and PLGA nanoparticles (160). In *D. melanogaster* models of PD, successful local delivery of curcumin to the brain via nanocomposites has included an alginate-curcumin nanostructure, dosed to flies at concentrations as low as 10^−5^ g/mL and a curcumin monoglucoside at 10 μ M (155, 164). A polymeric polyvinylpyrrolidone-curcumin nanoparticle, dosed between 5 μ M and 10 μ M was also successful at increasing the bioavailability of curcumin in *D. melanogaster* (165). In MPTP-treated mice, curcumin-loaded polysorbate 80-modified cerasome nanoparticles of about 110 nm in diameter had much greater solubility and BBB passage compared to free curcumin. Furthermore, their effectiveness was enhanced with ultrasound-targeted microbubble destruction (166).

### 3.4. Resveratrol

Resveratrol is a phelonic compound of the stilbenoid class. It is the most well-known phytochemical to provide anti-aging benefits and, therefore, has been widely studied for its antioxidant, anti-inflammatory, and senolytic properties. Resveratrol is found mainly in pigmented fruits and vegetables, such as grapes, blueberries, and cranberries, but it is also found in peanuts, cocoa, and wine (167). However, a recent study showed that resveratrol found in rice callus dramatically extended the life of *D. melanogaster* and prevented age-related tissue degeneration (168). The biosynthesis of resveratrol relies on the conversion of *p*-coumaric acid to *p*-coumaroyl CoA by 4-coumarate-CoA ligase. Thereafter, three malonyl-CoA molecules and one *p*-coumaroyl CoA molecule synthesize resveratrol through the enzymatic activity of stilbene synthase (169). Resveratrol has been shown to be neuroprotective in *D. melanogaster* models of PD, but its senolytic capacity is dynamic and complicated.

The therapeutic benefit of resveratrol has been studied in *parkin* loss-of-function mutated *D. melanogaster*. After a three-week period, the survival rate of untreated *parkin*-mutant flies was found to be 75% less than that of the wild type flies. However, a significant enhancement in survival rate was observed in the *parkin*-mutant flies that were treated with resveratrol (15, 30, and 60 mg/kg diet) compared to the untreated *parkin*-mutant flies (170). Additionally, in comparison to the resveratrol-treated *parkin* mutant flies, the untreated *parkin* mutant flies exhibited worse performance on the negative geotaxis assay. Concomitant with the climbing results, AChE activity in resveratrol-treated *parkin* mutant flies increased about 2.1, 2.4, and 2.5 folds for 15, 30, and 60 mg/ kg diet of resveratrol compared to the AChE activity in untreated *parkin* mutant flies. Furthermore, untreated *parkin* mutant flies showed a 3.4-fold decrease in AChE activity compared to wild type flies. The oxidative stress markers of H_2_O_2_, MDA, and NO were elevated in *parkin*-mutant flies compared to the wild type and reduced in untreated *parkin*-mutant flies compared to those treated with resveratrol. Resveratrol also increased the levels of non-protein and total thiols, which helped establish a redox balance in the *parkin* mutant *D. melanogaster*. Furthermore, compared to wild type flies, untreated *parkin* mutant *D. melanogaster* have downregulated *ple* and *Sod1* genes, which are responsible for encoding tyrosine hydroxylase and superoxide dismutase 1, respectively. However, these genes experienced upregulation in a resveratrol dose-dependent manner. Moreover, histology data showed no detectable brain lesions in the control or resveratrol-treated *parkin* mutated *D. melanogaster*. Finally, the mitochondrial mass in the brains of resveratrol-treated *parkin* mutated flies was significantly higher than the untreated *parkin* mutated flies and was comparable to wild type fly brains. Therefore, resveratrol is neuroprotective due to its antioxidant properties and ability to influence gene expression (170).

MPTP *D. melanogaster* models of PD constructed using 1,000 μ M -3,000 μ M MPTP experienced 100% mortality within a week (63). However, MPTP concentrations of 250 μ M and 500 μ M were not as lethal. Furthermore, MPTP treatment induced a pronounced reduction in climbing rates, the emergence of offspring, cell viability, AChE, catalase, and glutathione-S-transferase activity, eosinophilia, rarefaction of CNS white matter, segmental loss of CNS neurons, and increased H_2_O_2_ and NO levels compared to the control group. However, all these parameters were significantly ameliorated when resveratrol was co-administered with MPTP (63). Even in the absence of MPTP, resveratrol consumption throughout the lifecycle significantly increased wild type *D. melanogaster* lifespan. For example, when *D. melanogaster* were given resveratrol doses at 30 mg/kg and 60 mg/kg of diet, their lifespan lengthened by 39.5 and 41.9%, respectively (63). *D. melanogaster* lifespan extension by resveratrol has been shown to be dependent on the activity of sirtuins and their antioxidant and anti-inflammatory properties, rather than explicitly by senolytic activity (63, 171).

The resveratrol found in grape skin extract (GSE) is neuroprotective and effective at rescuing PD-related deficits, primarily through mitochondrial preservation. PINK1 mutant *D. melanogaster* experience abnormal mitochondrial aggregation in their flight muscles, which leads to flight muscle degeneration and subsequent aberrant wing positioning (172). However, moderate to high quantities of GSE (8 and 16% of food, respectively) amplified the flight muscular production of ATP, reduced ROS generation, reduced mitochondrial aggregation in flight muscles, improved wing posture, and ameliorated the locomotor activity deficit of the PINK1 mutant *D. melanogaster* PD model (172). Moreover, the 16% GSE diet concentration proved effective in preventing mitochondrial aggregation in DA neurons and DA neuron loss in the PINK1 mutant flies (172). Furthermore, GSE treatment of PINK1 mutants reduced p62 accumulation, induced autophagy, increased LC3-I to LC3-II conversion, and restored C-I 30 protein levels. GSE had contrasting effects in wild type flies, whereby p62 and LC3 expression was increased without affecting the LC3-II/LC3-I ratio (172). These effects indicate that the anti-aging properties of GSE are attained through improving overall mitochondrial integrity, activation of mitophagy, enhanced autophagy, and the increased expression and deployment of autophagy receptors (172).

*D. melanogaster* chronically exposed to 100 μM of rotenone every 10 days experience age-related and rotenone-related reduced survival rate, locomotion impairment, loss of DA neurons and TH proteins, and increased expression of *dSarm*, the *D. melanogaster* homolog of Sarm1 (173). *dSarm* promotes axonal degeneration, triggers inflammation, and is necessary and sufficient for rotenone-induced locomotor deficits (173, 174). Interestingly, the locomotor deficits caused by rotenone are independent of elevated levels of ROS (173). Instead, resveratrol mitigated the inflammation response to rotenone treatment and rescued the associated locomotor deficits by downregulating *dSarm* expression (170). Therefore, *dSarm* is a key pro-inflammatory mediator of rotenone-induced neurotoxicity (173).

Despite the focus of these *D. melanogaster* studies on resveratrol’s antioxidant, anti-inflammatory, and senomorphic mechanisms for neuroprotection, other studies have focused on its senolytic capability, its harmful effects, or even its benign influence on health (90). Although the delineation is somewhat arbitrary, smaller resveratrol doses of less than 10 μ M are sufficient to yield its senomorphic antioxidant behavior (90). For example, in the aforementioned study, resveratrol was fed as a dietary supplement at a low dose of either a 30 mg/kg diet or a 60 mg/kg diet (63). Furthermore, at 6 μ M, resveratrol was neuroprotective against a *C. elegans* model of Alzheimer’s disease by reducing protein aggregation (175). Other actions, when taken together, could straddle between being senomorphic, anti-senescent, and fully senolytic, such as the activation of telomerase, activation of Silencing Information Regulator 2-Related Enzyme 1 (SIRT1), inhibition of NF-kB, and upregulation of Nrf2 signaling (176–179).

However, there is evidence that resveratrol at higher doses can act as a senolytic, pro-oxidant, or even induce senescence (90, 180, 181). In a recent study, 100 μM of resveratrol seemingly acted as a senolytic in rat primary culture models of intervertebral disc degeneration (180). For example, compared to the untreated diseased state, resveratrol increased the number of proliferative cells, decreased the number of SA-β-Galstaining-positive cells, restored the imbalance between the number of cells in the G0/G1 and S phases, increased telomerase activity, and reduced the expression of the p16 and p53 genes (180). However, at high concentrations, resveratrol can be cytotoxic (90, 181). There have also been mixed results in clinical studies (182–185).

The current consensus on the use of resveratrol is that the compound has well-established senomorphic properties for a variety of pathologies, but there are other possible outcomes of its use (186). More research is needed to fully explore its ability to act as a senolytic. Despite the enigmatic behavior of resveratrol, it is currently used in many clinical trials and is generally well tolerated. Therefore, it is possible to get past the preclinical stage of investigation for PD, but likely will require bioengineering improvement (187, 188).

### 3.5. Piperlongumine

Piperlongumine (PLG) is a senolytic alkaloid commonly derived from the long pepper plant *Piper longum* Linn. *P. longum* is native to the tropical rainforests in South Asia, including those in India, Malaysia, Nepal, Sri Lanka, and Vietnam, among other countries (189). The biosynthesis of PLG is not well characterized, but there have been many successful efforts to develop synthetic PLG analogs to study, improve, and market its pharmacological properties (190–193). It boasts impressive anticancer properties in addition to antioxidant, anti-inflammatory, neuroprotective, and senolytic characteristics (194). It has been used as a spice and a medicinal elixir for millennia. In fact, it has been written about in detail by Hippocrates in Greece, used in India’s Ayurveda, and is included in traditional Chinse medicine (195, 196).

Several senolytic mechanisms of PLG have been established. For example, in WI-38 senescent cells, PLG selectively binds to the protein oxidation resistance 1 (OXR1), targeting it for proteasomal destruction via the ubiquitin-proteasome system (166). During periods of genome instability that lead to a DNA damage response, OXR1 maintains cell survival by activating G2-phase cell cycle arrest and inhibiting oxidative stress (197). Therefore, upregulated OXR1 is proposed as the mechanism that confers oxidative stress resistance to senescent cells. Conversely, suppression of OXR1 by PLG binding or by genetic knockdown in senescent cells induces apoptosis through reduced antioxidant enzymatic activity and increased ROS levels (197, 198). However, the senescent cell apoptosis pathway induced by PLG is ROS-independent. Instead, it depends on activated caspase-3 and the degradation of poly (ADP-ribose) polymerase (199).

PLG is neuroprotective beyond its senolytic abilities. For example, PLG activates the transcription factor Nrf2 to regulate cellular oxidative and inflammation homeostasis (200). Furthermore, PLG analogs have been manufactured to exert antioxidant and anti-inflammatory effects via Nrf2 activation (191). In a transgenic α-Syn *D. melanogaster* model, the genetic overexpression of Nrf2 or the overexpression of Nrf2’s dimerization partner, Maf-S, restores the α-Syn associated locomotion impairments and dopaminergic neuron degeneration (201). Conversely, reduced expression of the main Nrf2 inhibitor, Keap1, also protects against dopaminergic neuron loss in the transgenic α-Syn *D. melanogaster* model of PD (201). Furthermore, motor behavior assessments using rotarod and pole tests revealed that both PLG and L-DOPA ameliorated rotenone-induced motor deficits in mice and maintained tyrosine hydroxylase (TH) and dopamine levels (202).

PLG is also involved in restoring the impaired balance between autophagy and apoptosis that is characteristic of PD (203). In rotenone-induced SK-N-SH cell and mouse models of PD, PLG promotes autophagy and suppresses apoptosis to reverse the apoptotic-dominant nature of degenerative neurons in PD. PLG increases the rate of autophagy, as indicated by the increased conversion rate of cytosolic microtubule-associated Protein 1 Light Chain 3 alpha-I (LC3B-I) to the lipid-conjugated form, LC3B-II. This effect continued even when combined with bafilomycin A1, a lysosome inhibitor. Live-cell imaging confirmed that PLG induces autophagy and clears damaged mitochondria. Finally, PLG separates the heterodimer between the B-cell lymphoma 2 protein (BCL2) and the Beclin-1 protein, an activity that leads to autophagy. It also underscores BCL2’s involvement in PLG’s autophagy-promoting attributes (202). Additionally, PLG suppresses apoptosis by counteracting the rotenone-induced loss of mitochondrial membrane potential, blocking the mitochondrial permeability transition pore and preserving the function of mitochondrial complex I (202). Similarly, PLG reduces the extent that rotenone activates the pro-apoptotic proteins caspases-3 and caspases-9. Finally, PLG’s role in the autophagy-apoptosis balance is facilitated by the increase of BCL2 phosphorylation at Ser70 via MAPK8 signaling.

The PD-relevant neuroprotective and senolytic properties of PLG have been studied in SCNA transgenic SK-N-SH cells, senescent WI-38 human fibroblasts, 6-OHDA treated PC12 cells, rotenone-treated SK-N-SH cells, rotenone-treated mice, MPTP treated rats, 6-OHDA treated rats, and in lipopolysaccharide treated BV2 microglial cells (192, 199, 202, 204–206). Notably, it has not yet been studied in *D. melanogaster*. Furthermore, all PD models used to study PLG except for the SCNA transgenic SK-N-SH cells have been acute toxin models. Acute toxin models are efficient to execute but do not lead to the progressive accumulation of α-Syn and Lewy bodies formation, which are the two cardinal histological signs of PD (207, 208). In contrast, genetic models allow for the replication of specific disease aspects in isolation and can be used to study the early stages of PD development and progression (209). However, genetic models often do not exhibit hallmark clinical manifestations such as tremors, bradykinesia, and non-motor symptoms. The complications associated with various model organisms underscores the value of utilizing a variety of *in vitro*, *in vivo*, and *in silico* approaches to study pathophysiology and drug development (210). *D. melanogaster* is a paramount model for studying the roles of genetics in PD and should be used in the characterization of PLG as a neuroprotectant and senolytic.

**Figure 1:**
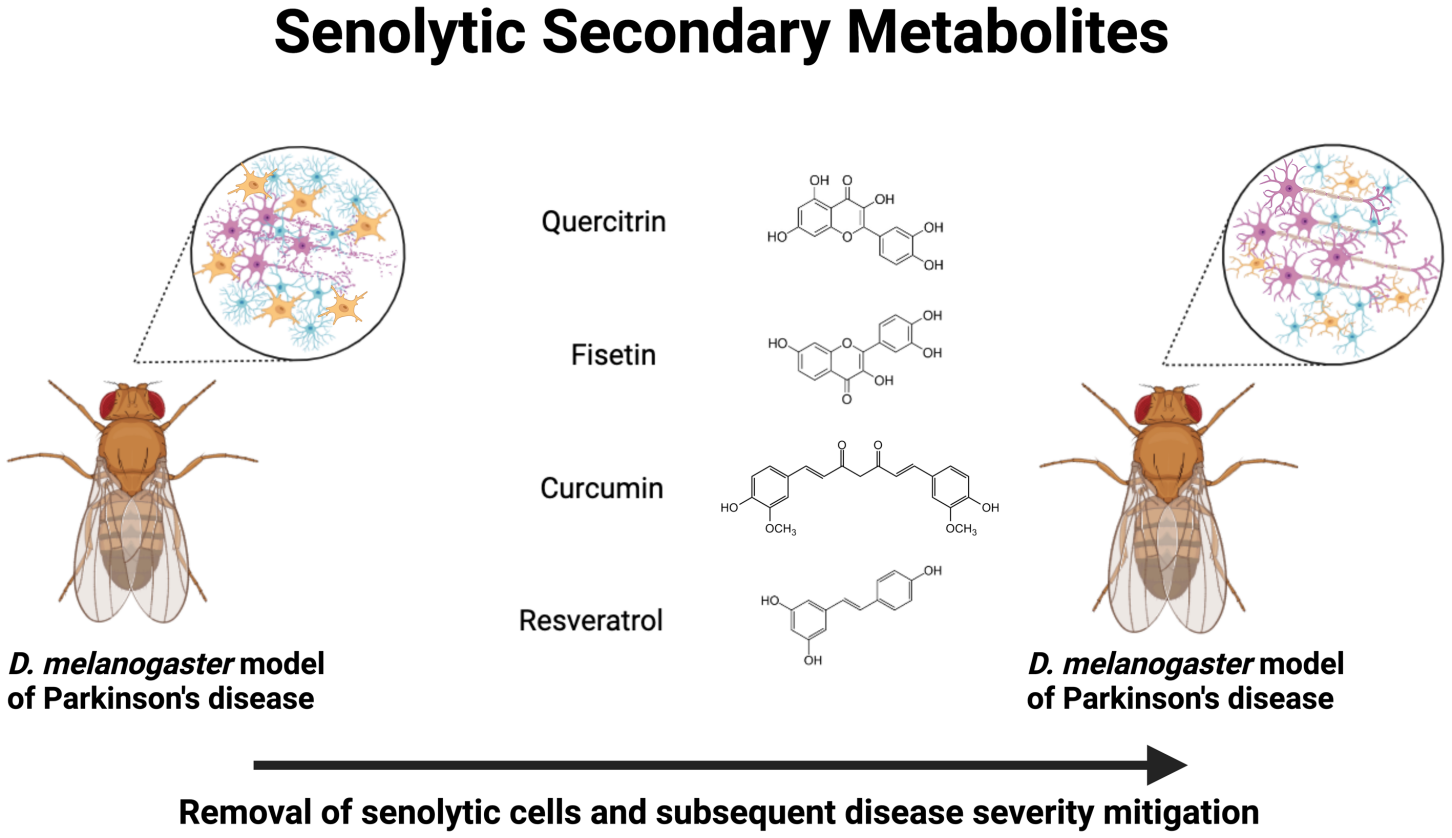
Neuroprotective senolytic secondary metabolites in *Drosophila melanogaster* models of Parkinson’s disease. Treatment with quercitrin, fisetin, curcumin, and resveratrol exert a senolytic effect, which mitigates neurodegenerative pathophysiology. Created with BioRender.com

*D. melanogaster* is an efficient model for screening secondary metabolites, facilitating the identification of potential therapeutic candidates, and for the elucidation of molecular mechanisms of action. Further research is needed to clarify their precise mechanisms of action and evaluate their efficacy, safety, and potential for therapeutic applications in age-related diseases. In conclusion, secondary metabolites offer a promising avenue for the development of senolytic agents. These compounds interact with intracellular targets, modifying cellular behavior and selectively eliminating senescent cells. By targeting senescent cells and addressing the detrimental effects of cellular senescence, secondary metabolites hold potential as therapeutic interventions for age-related diseases. The information presented in this section is summarized in Table 1 and a schematic is presented in Figure 1.

**Table 1:** The molecular, cellular, and phenotypical effects of senolytic secondary metabolites in various *D. melanogaster* models of Parkinson’s disease.

Metabolite	Model	Citation	Effects on cellular and molecular parameters	Effects on phenotypical parameters
Quercetin	Rotenone	(102)	↑ Mitochondrial complex I through IV activity	↑ Lifespan, health span
			↓ Autophagy signaling, UPR signaling, cytokines	↓ Motor deficit
	Blue light	(110)	↓ Senescent cells, male fly protein content, female fly lipid content	↑ Lifespan, heat stress survival, male activity, egg production
	H_2_O_2_	(111)	↓ Hsp70, Upd1, protein carbonyls, thiobarbituric reactive substances	↑ Negative geotaxis performance
			↑ Superoxide dismutase, glutathione, catalase quantity	
	Paraquat	(116)	↑ Superoxide dismutase, glutathione, catalase quantity	↑ Lifespan, negative geotaxis performance
			↓ ROS, lipid peroxidization	
Fisetin	Paraquat	(125)	↑ Glutathione, catalase, superoxide dismutase quantity	↑ Lifespan, negative geotaxis performance
			↓ ROS, acetylcholine esterase activity	
Curcumin	Paraquat	(144)	↓ Gene expression, dopaminergic degeneration	↑ Lifespan
	Alpha synuclein	(146)	↑ Oligomer root-mean-squared deviation and radius of gyration	↓ Alpha synuclein fibrillation and aggregation into Lewy bodies
			↓ Oligomer beta-sheet content and backbone hydrogen bonds	
	dUCH knockdown	(154)	↓ ROS, preserved dopaminergic neuron quantity	↑ Crawling speed, coordination, negative geotaxis performance
	Rotenone	(155)	↑ Glutathione, dopamine, and dopamine metabolite quantity	↑ Lifespan, negative geotaxis performance
			↓ ROS, hydroperoxides	
	Rotenone	(156)	↓ ROS, caspase-3 activity, caspase-9 activity	↑ Lifespan, negative geotaxis performance
			↑ Dopaminergic neuron quantity	
	Alpha synuclein	(157, 158)	↓ ROS, lipid peroxidization, protein carbonyl, apoptosis	↑ Lifespan, health span
	LRRK2 & H_2_O_2_	(159)	↓ Dopaminergic neuron loss, brain oxidized proteins, LRRK2 kinase activity	↑ Lifespan, negative geotaxis performance
Resveratrol	PARK	(170)	↑ AChE activity, thiol quantity, *ple* and *Sod1* expression, mitochondrial brain mass	↑ Lifespan, negative geotaxis performance
			↓ H_2_O_2_, MDA, NO, brain lesions	
	MPTP	(63)	↑ Offspring, activity of AChE, catalase, glutathione-S-transferase, eosinophilia, rarefaction of CNS white matter, CNS neuron quantity	↑ Lifespan, negative geotaxis performance
			↓ H_2_O_2_ and NO	
	PINK1	(172)	↑ ATP production in flight muscles, wing posture, autophagy, LC3-I to LC3-II conversion, C-130 protein levels	↑ Rate of flying and jumping events
			↓ ROS, mitochondrial aggregation in flight muscles and dopaminergic neurons, dopaminergic neuron loss, p62 accumulation	
	Rotenone	(173)	↓ Inflammation, *dSarm* expression	↑ Lifespan, negative geotaxis performance

## 4. Discussion

L-DOPA is the primary therapy for PD, but its prolonged use leads to drug-induced dyskinesia. Other drug strategies, like dopamine agonists and catechol-O-methyltransferase inhibitors, have non-motor side effects such as hallucinations, constipation, and orthostatic hypotension. Surgical options like deep brain stimulation improve motor dysfunction, but its efficacy drops over time as it does not address the neurodegeneration. Therefore, there is a clear and pressing need for more effective therapeutics, in addition to earlier intervention facilitated by a pre-symptomatic diagnosis.

A diet rich in fruits and vegetables has protective properties against chronic diseases. Additionally, plant-derived products have been crucial in drug development. In drug discovery from plant sources, concerns about “dirty” molecules interacting with multiple protein targets are common. These interactions can be noxious, activate cellular stress response pathways, increase lifespan, and enhance the survival of the organism that consumed the stressed plant. The concept of hormesis illustrates how low doses of these phytochemicals can offer beneficial effects, while high doses may result in toxicity. The intermittent and moderate activation of stress response pathways by these potentially harmful phytochemicals could play a key role in delivering cellular protection. In a similar fashion, periodic shifts in metabolic activity, like those seen in intermittent fasting, can prolong lifespan and offer neuroprotection due to the temporary triggering of stress response pathways by ketones generated from fatty acids. Because these noxious phytochemicals can impact evolutionarily conserved cellular signaling pathways, it is possible to extend these discoveries from invertebrate to mammalian models of diseases.

Secondary plant metabolites can influence evolutionarily conserved pathways, offering potential neuroprotection against PD by activating adaptive cellular stress responses. One such pathway is the Nrf2 / antioxidant response element (ARE) pathway, which regulates antioxidants and detoxifying enzymes in response to phytochemicals. Activation of the Nrf2 signaling pathway, either through genetic manipulation or through secondary metabolites like quercetin, PLG, or sulphorafane, is neuroprotective in *D. melanogaster* PD models (201, 211, 212). The transcription factor NF-kB controls genes related to immunity, inflammation, stress response, cell survival, and proliferation. As such, it has been described as a “master regulator of evolutionarily conserved biochemical cascades” (213). In PD and other neurodegenerative diseases, NF-kB activity is compromised. However, secondary metabolites like quercetin and resveratrol have been shown to restore NF-kB function and offer neuroprotection in *D. melanogaster* models of PD (116, 214). Sirtuins, such as SIRT1, are highly conserved NAD + -dependent deacetylases that regulate key transcription factors (215, 216). Activation of SIRT1 via secondary metabolites, such as resveratrol and quercetin, confers neuroprotection in *D. melanogaster* models of PD (217). *D. melanogaster* has been a valuable model for studying potential drug targets in shared molecular pathways between humans and flies that can be validated in vertebrates.

Cell culture-based screening in drug discovery has limitations in mimicking *in vivo* responses, leading to ineffective or toxic results when validated in animal models. Although 3D cell cultures better replicate *in vivo* responses, they are costly for high-throughput screening. Rodent models, while useful, are expensive and time-consuming for initial drug screening. On the other hand, *D. melanogaster*, with its highly conserved molecular pathways relevant to human diseases, provides a cost-effective *in vivo* model for large-scale screening of phytochemicals targeting PD. *D. melanogaster* models offer powerful molecular tools for gene manipulation, making them ideal for screening bioactive compounds. Their genetic flexibility and cost-effectiveness compared to rodents make them valuable for evaluating drug candidates. The short life cycle and behavioral assays of *D. melanogaster* allow swift screening of numerous candidates, including those targeting PD-related mobility defects. Utilizing *D. melanogaster* models as an *in vivo* screening platform overcomes the limitations of *in vitro* assays, enabling rapid identification of potential drug candidates for further validation in mammalian models. The success of *D. melanogaster* in screening for diseases highlights its potential as a cost-effective solution in early-stage drug discovery.

*D. melanogaster* models, like any model system, have some disadvantages to consider. While they exhibit a high degree of conservation in molecular signaling pathways relevant to disease, there are notable physiological differences, including brain anatomy and BBB permeability. Additionally, *D. melanogaster* lacks a classical adaptive immune response, limiting the study of neuroinflammation relevant to humans. However, *D. melanogaster* offers insight into the innate immune response, contributing to disease pathogenesis without interference from adaptive immune signaling. Other drawbacks include the need for frequent passaging, the inability to freeze *D. melanogaster* strains, and the presence of vertebrate-specific factors linked to disease pathology. Despite these limitations, *D. melanogaster* remains a powerful model system, serving as an effective screening platform for drug discovery and enabling the development of novel assays to understand the mechanistic effects of bioactive compounds on disease pathogenesis.

*D. melanogaster* with mutated vesicular monoamine transporters (dVMAT) have successfully been used to identify, characterize, and validate drugs that rescue motor behavior in PD, namely pergolide and dacarbazine (12, 218). Pergolide is a dVMAT-independent dopamine agonist that acts on the CNS to increase dopamine levels. Dacarbazine is a dVMAT-dependent anticancer drug that has shown potential in treating PD by promoting neuroprotection and neuroregeneration. Both of these drugs have been confirmed to be aminergic and able to improve locomotion in *D. melanogaster* that have either weakly expressed or null dVMAT (218).

Pergolide improves symptoms in PD patients, either as a standalone treatment or in combination with levodopa (219, 220). The symptomatic improvement of pergolide is attributed to its action on both dopamine D1 and D2 receptors, whereas other drugs, like bromocriptine, primarily target only dopamine D2 receptors (219). However, the use of pergolide has also raised concerns regarding its potential side effects. Studies have concluded that PD patients treated with ergot-derived dopamine agonists, including pergolide and cabergoline, may be at an increased risk of developing cardiac valvulopathy by activating serotonin receptors in cardiac valves, leading to fibrosis and valve dysfunction (221, 222). Therefore, pergolide has been withdrawn from the U.S. market (223).

Dacarbazine, also known as DTIC-Dome, has traditionally been used to treat malignant melanoma and Hodgkin’s disease. In rodent models, it has been shown to exhibit antioxidant and anti-inflammatory effects (224). Interestingly, a PD patient’s response to levodopa improved while receiving dacarbazine as part of their treatment for melanoma (225). However, dacarbazine has also been shown to cause mitochondrial dysfunction and promote oxidative stress in rat hepatocytes (226). Due to the inconsistency of pre-clinical results, dacarbazine has not yet been brought into the mainstream drug arsenal for treating PD patients.

The translational potential of *D. melanogaster* models in PD research is evident, as they have facilitated the evaluation of potential therapeutic compounds, allowing for the screening and identification of novel drug candidates (12). These findings emphasize the significance of *D. melanogaster* in PD-related therapeutic research, as it continues to play a vital role in advancing the understanding of the disease and aiding in the development of potential therapeutic interventions. Despite the progress, however, current PD treatments remain palliative, primarily focused on symptom relief rather than addressing the disease’s root cause, with common medications such as L-DOPA and dopamine agonists often leading to adverse side effects over the long term (227). There is an urgent need for innovative therapeutic approaches that target the underlying causes of PD. Emerging research has explored gene therapy, stem cell transplantation, deep brain stimulation, microbial treatment, and neuroprotective agents (228). Particularly, the focus has shifted towards targeted drug delivery systems that can deliver therapeutic agents specifically to the brain regions affected, thereby enhancing treatment efficacy while minimizing side effects. Given the well-characterized anatomical and behavioral simplicity of *D. melanogaster* and their long-established role as PD models, their potential in shaping these advanced treatments appears promising.

## Author contributions

SM: Investigation, Writing – original draft, Writing – review & editing. RD: —. SW: Investigation, Writing – original draft, Writing – review & editing. JL: Investigation, Writing – original draft, Writing – review & editing. RL: Conceptualization, Investigation, Supervision, Writing – original draft, Writing – review & editing.

## Glossary

6-OHDA: 6-Hydroxydopamine
α-Syn: Alpha-synuclein
ARE: Antioxidant response element
BBB: Blood–brain barrier
BCL2: B-cell lymphoma 2 protein
CNS: Central nervous system
*D. melanogaster*: *Drosophila melanogaster*
DAT: Dopamine membrane transporters
dVMAT: Drosophila vesicular monoamine transporter
ERK: Extracellular signal-regulated kinase
GBA: Glucocerebrosidase
GSE: Grape skin extract
GSHJ: Golden-silkworm Hongjam
L-DOPA: Levodopa
LC3B-I: Cytosolic microtubule-associated Protein 1 Light Chain 3 alpha-I
LID: Levodopa-induced dyskinesia
MPP+: 1-Methyl-4-phenylpyridinium
MPTP: 1-Methyl-4-phenyl-1,2,3,6-tetrahydropyridine
NAC: Non-amyloid-β-component
NADPH: Nicotinamide adenine dinucleotide phosphate
NAT: Noradrenaline membrane transporters
Nrf2: Nuclear factor erythroid 2-related factor 2
*OXR1*: Oxidation resistance 1
*P. longum*: *Piper longum* Linn
Paraquat: 1,1′-Dimethyl-4,4′-bipyridinium dichloride
PD: Parkinson’s disease
*PINK1*: PTEN Induced Kinase 1
PLG: Piperlongumine
*PRKN*: Parkin RBR E3 ubiquitin protein ligase
ROS: Reactive oxygen species
SASP: Senescence-associated secretory phenotype
SIRT1: Silencing Information Regulator 2-Related Enzyme 1
*SNCA*: Synuclein Alpha
SNpc: Substantia nigra pars compacta
SOD: Superoxide dismutase
TH: Tyrosine hydroxylase
VPS35: Vacuolar protein sorting 35
